# Kaolin Clay Anemia

**DOI:** 10.7759/cureus.13796

**Published:** 2021-03-10

**Authors:** Barrett O Attarha, Sebastian Mikulic, Ciel Harris, James S Scolapio

**Affiliations:** 1 Internal Medicine, University of Florida College of Medicine, Jacksonville, USA; 2 Gastroenterology, University of Florida College of Medicine, Jacksonville, USA

**Keywords:** anemia, pica, geophagia, iron deficiency anemia

## Abstract

Iron deficiency anemia is a common diagnosis encountered in the nutrition, primary care, and gastroenterology fields. Iron deficiency anemia most often leads to evaluation for various malabsorption disorders and colonoscopy to exclude colon cancer as an etiology. We present a case of iron deficiency anemia that was caused by geophagia. After the culprit dietary habit was stopped, the patient's iron deficiency anemia subsequently resolved.

## Introduction

Iron deficiency is the most common cause of anemia worldwide. Some common causes include dietary deficiency, malignancy, decreased absorption (autoimmune gastritis, celiac sprue, *Helicobacter pylori*, gastritis, zinc deficiency), blood loss (gastrointestinal, menstrual), and increased requirements (pregnancy, lactation). Primary symptoms include palpitations, fatigue, dizziness, tachycardia, and dyspnea on exertion. Severe deficiency may cause a smooth tongue, koilonychia, and cheilosis. Laboratory findings will include low mean corpuscular volume (MCV), low or inappropriately normal reticulocyte count, decreased ferritin, decreased serum iron levels, and elevated transferrin levels leading to decreased transferrin saturation. As the MCV falls, blood smear will reveal hypochromic microcytic cells, anisocytosis (variation in red blood cell (RBC) size), and poikilocytosis (variation in RBC shape). Differential diagnosis includes anemia of chronic disease, thalassemia, lead poisoning, and congenital X-linked sideroblastic anemia. Management focuses on the replacement of iron stores, and perhaps more importantly, identification of the cause [1].

An important association of iron deficiency includes pica, or craving for specific foods of no nutritional value. It is unclear whether pica is the result of iron deficiency or vice versa. In one study of 55 patients with iron deficiency anemia in the setting of gastrointestinal blood loss, pica was present in 32 patients (58%) in the form of pagophagia, or craving of ice [2]. However, there are also reports in the medical literature that support that the ingestion of certain substances like clay, known as geophagia, may contribute to iron-deficiency anemia. Kaolinite, a component of kaolin clay, reduces iron absorption in the duodenum [3]. Therefore, ingestion of large amounts of kaolin over time can lead to an iron-deficient state. It is important for medical practitioners to understand and recognize this association as numerous diagnostic tests can be averted with a simple history. In this case, we present a patient who developed iron-deficiency anemia after consuming kaolin clay for multiple years. 

## Case presentation

A 47-year-old African American male was admitted to the hospital from the emergency department with a two-week history of epigastric abdominal pain, headaches, light-headedness with pre-syncope, and intermittent constipation. The patient had a past medical history significant for Diabetes Mellitus Type II (controlled on metformin), hypertension, and obesity. He also had intermittent joint pain treated with an oral nonsteroidal anti-inflammatory drug (NSAID). The patient denied any melena, hematemesis, or bloody stools. Physical exam was significant for a BMI of 50 and generalized epigastric tenderness on palpation. Rectal exam was guaiac positive for blood. At presentation, laboratory tests were significant for microcytic anemia with a hemoglobin of 7.0 (normal range for males 14-18 g/dl), low serum iron of 12 (normal range, 32-159 ug/dl), and a low serum ferritin of 8.2 (normal range, 30-400 ng/ml) (Table 1). Serum electrolytes were grossly normal. Computer tomography of the abdomen was significant for a large hiatal hernia and otherwise unremarkable. The gastroenterology service was consulted by the internal medicine service for possible intestinal bleeding. Based on the patient’s clinical history of epigastric abdominal pain, guaiac positive stool, and NSAID use, an upper endoscopy was performed. 

**Table 1:** Hematology results at presentation, day 60, and one year after discontinuation of clay consumption Caption: HGB: hemoglobin, RBC: red blood cell count, HCT: hematocrit, MCV: mean corpuscular volume, MCH: mean corpuscular hemoglobin, MCHC: mean corpuscular hemoglobin concentration, RDW: red cell distribution width

Tests	Presentation	Day 60	1 year
HGB (14-18 g/dl)	7.0	9.2	14.2
RBC (4.5 x 6.3x10E6 UL)	4.41	5.16	4.61
HCT (40-54%)	26.5	33.2	42.1
MCV (82-101 fL)	60.1	64.3	91.3
MCH (27-34 pg)	15.9	17.8	30.8
MCHC (31-36 g/dl)	26.4	27.7	33.7
RDW (12-16%)	23.7	26.8	12.3

The exam was significant for a large hiatal hernia without Cameron erosions and otherwise normal with no bleeding source seen. Small intestine biopsies were taken from the duodenum to exclude celiac disease. The biopsy results were later reported as normal. The patient had a colonoscopy two years prior to this presentation which was positive for large internal hemorrhoids and was otherwise normal. A colonoscopy was therefore not performed on this hospital admission. On further questioning, it was discovered that the patient was consuming Kaolin white clay for the past two years. Two years prior to consumption of the clay his hemoglobin was recorded in the medical record as normal (Table 1). The patient also admitted to the craving for ice over the past year. The patient noted that he began eating clay after watching a 2016 PBC documentary titled *Eat White Dirt* [4]. The patient had no medical history of an eating disorder and there was no documented obsessive-compulsive disorder, although he did mention occasional binge eating. 

## Discussion

This case study illustrates four key points. First, the importance of asking patients about dietary habits including the consumption of oral supplements that may explain their clinical presentation; in this case, the consumption of clay resulting in iron deficiency anemia. Second, it’s important to have a broad differential diagnoses when evaluating a patient with iron deficiency anemia. It is a common clinical mistake to contribute all cases of iron deficiency anemia to gastrointestinal blood loss without exploring other possible etiologies as listed in Table 2. In this case, the patient’s internal hemorrhoids can explain the guaiac positive stool given an otherwise normal quality colonoscopy exam two years prior. We have found it helpful to make it a routine habit of asking questions related to Table 2 in every patient presenting with iron deficiency anemia, specifically those causes marked with an asterisk. This can help lead to the correct diagnosis as occurred in this case. Third, there is some uncertainty of what comes first, iron deficiency followed by pica or pica that then results in iron deficiency. In this specific case we have documented that iron-deficiency occurred after the consumption of clay and resolved after the consumption was discontinued. The various different types of pica are also listed for reference in Table 3. Finally, a knowledge of geophagia and its potential medical complications is helpful for all medical providers evaluating patients with nutrient deficiencies. In this case, the deficiency was specific to iron.

**Table 2:** Causes of iron deficiency anemia unrelated to gastrointestinal bleeding * It helps to make it a routine habit of asking questions related to Table 2 in every patient presenting with iron deficiency anemia, especially those causes marked with an asterisk.

Blood loss	Reduced absorption	Redistribution after bone marrow stimulation	Hemosiderosis	Diet	Hemolysis	Drug-related
Traumatic hemorrhage, Hemoptysis, *Menorrhagia, Pregnancy/Delivery, Hematuria, *Frequent blood donation, Excessive blood draws, Lactation	*Celiac disease, Gastritis (Autoimmune, Helicobacter pylori), Extensive mucosal injury to GI tract (Inflammatory bowel disease, bacterial overgrowth, Whipple’s disease), *Bariatric and gastric bypass surgeries	Hypo-response to erythropoietin (End-stage renal disease)	Urinary, Pulmonary	*Vegan diet, Malnutrition, Limited diet, *Supplements (clay)	Prosthetic valves, PNH (paroxysmal nocturnal hemoglobinuria), Marathon runners, Hemoglobinopathies	Bone marrow suppressing medication (chemotherapy), PPI (proton pump inhibitors), Calcium supplements, Salicylates

**Table 3:** Types of pica

Type	Substance
Amylophagia	Starch and paste
Coprophagia	Feces
Geophagy	Soil, clay or chalk
Hematophagy	Blood
Hyalophagia	Glass
Pagophagia	Pathological consumption of ice
Self-cannibalism	Human body parts
Trichophagia	Hair or wool
Urophagia	Urine
Xylophagia	Wood
Cautopyreiophagia	Burnt match heads

Geophagia refers to patients that consume nonfood substances from the earth. While surprising in Western society, geophagia is an ancient practice that is still practiced in modern times for cultural or religious reasons [5]. Kaolin white clay, or kaolinite, is a common dirt found on the East Coast in the United States. This practice is deeply rooted in Southern culture in the United States, particularly in African American populations, and some anthropologists believe it was brought to the United States by the African slave industry [6,7]. The practice is often handed down from generation to generation. In these circumstances, consumption of clay would not be classified as a psychological form of pica based on the latest Diagnostic and Statistical Manual of Mental Disorders, Fifth Edition (DSM-5) classification (see Appendices). There is a belief among some that clay has health benefits such as helping with morning sickness, binding toxins, strengthening immune function, and helping with indigestion and diarrhea. In modern times, white clay is still available to purchase online at major retailers such as Amazon.

There are also obvious health risks in the consumption of soil, in particular, ingestion of helminth eggs, such as Ascaris and hookworm. Severe constipation and colon rupture have also been reported with clay consumption [8]. One study examined the effects of kaolin ingestion on pregnant rats and their fetal development. The results showed that rats who consumed kaolin had reductions in their hemoglobin, hematocrit, and RBC levels [9]. It is thought by some individuals that iron deficiency anemia causes pica. However, as illustrated in this case, it is more likely that the kaolin itself caused the iron deficiency anemia. It has also been hypothesized that kaolin clay creates iron deficiency anemia by preventing the absorption of iron in the gut, given its chemical composition [3]. Dirt and kaolin contain negatively charged cation-exchange sites that have the potential to bind iron given its positive charge, thus preventing intestinal absorption [10]. Although one can argue the patient’s history of NSAID use may predispose to gastrointestinal bleeding and, therefore, iron deficiency anemia, the patient denied any recent use over the past year. Additionally, in regards to the patient’s internal hemorrhoids, repeat guaiac testing on hospital admission prior to discharge was negative. It was concluded the cause of iron deficiency was likely secondary to kaolin ingestion.

## Conclusions

In this patient, after discontinuation of the consumption of clay, all symptoms resolved and he noted improved overall energy and wellbeing. Laboratory testing was normal when repeated approximately 12 months later. The patient also noted that it was easy for him to stop clay consumption after he was counseled that the clay was the cause of his iron deficiency anemia. Although uncommon, strange dietary habits can be responsible for nutritional deficiencies.

## Appendices

DSM-5 criteria for diagnosing pica [11]

- Persistent eating of non-nutritive substances for a period of at least one month

- The eating of non-nutritive substances is inappropriate for the developmental level of the individual

- *The eating behavior is not part of a culturally supported or socially normative practice

- If occurring in the presence of another mental disorder or during another medical condition, it is severe enough to warrant independent clinical attention
